# A Novel Role of Peripheral Corticotropin-Releasing Hormone (CRH) on Dermal Fibroblasts

**DOI:** 10.1371/journal.pone.0021654

**Published:** 2011-07-13

**Authors:** Olga Rassouli, George Liapakis, Iakovos Lazaridis, George Sakellaris, Kostas Gkountelias, Achille Gravanis, Andrew N. Margioris, Katia P. Karalis, Maria Venihaki

**Affiliations:** 1 Department of Clinical Chemistry, School of Medicine, University of Crete, Heraklion, Crete, Greece; 2 Department of Pharmacology, School of Medicine, University of Crete, Heraklion, Crete, Greece; 3 Department of Pediatric Surgery, University Hospital of Heraklion, Crete, Greece; 4 Developmental Biology Section, Biomedical Research Foundation of the Academy of Athens, Athens, Greece; 5 Division of Endocrinology, Children's Hospital, Boston, Massachussets, United States of America; New Mexico State University, United States of America

## Abstract

Corticotropin-releasing hormone, or factor, (CRH or CRF) exerts important biological effects in multiple peripheral tissues via paracrine/autocrine actions. The aim of our study was to assess the effects of endogenous CRH in the biology of mouse and human skin fibroblasts, the primary cell type involved in wound healing. We show expression of CRH and its receptors in primary fibroblasts, and we demonstrate the functionality of fibroblast CRH receptors by induction of cAMP. Fibroblasts genetically deficient in *Crh* (*Crh−/−*) had higher proliferation and migration rates and compromised production of IL-6 and TGF-β1 compared to the wildtype (*Crh+/+*) cells. Human primary cultures of foreskin fibroblasts exposed to the CRF_1_ antagonist antalarmin recapitulated the findings in the *Crh−/−* cells, exhibiting altered proliferative and migratory behavior and suppressed production of IL-6. In conclusion, our findings show an important role of fibroblast-expressed CRH in the proliferation, migration, and cytokine production of these cells, processes associated with the skin response to injury. Our data suggest that the immunomodulatory effects of CRH may include an important, albeit not explored yet, role in epidermal tissue remodeling and regeneration and maintenance of tissue homeostasis.

## Introduction

Wound healing is a highly coordinated, dynamic, and interactive process aiming to repair the injury and restore the functional integrity of the wounded tissue. Following skin injury, different cell types interact to initiate a sequence of events that includes coagulation, inflammation, and formation of granulation tissue, re-epithelialization and finally remodeling [1]. Dermal fibroblasts are critical cells in this process through their proliferation, ordered migration into the provisional matrix, production of extracellular matrix and differentiation into myofibroblasts [2]. The above together with the fibroblast-mediated effects on keratinocyte proliferation, differentiation, and migration place these cells in a critical position for re-epithelialization and preservation of epidermal homeostasis after tissue injury [3].

Interleukin (IL)-6 and other proinflammatory cytokines and growth factors, produced locally in both human and murine skin cells and resident immune cells, is a major regulator of the healing process. IL-6 is a pleiotropic cytokine involved in the growth and differentiation of numerous cell types including those of dermal and epidermal origin [4]. In the skin IL-6 is produced primarily by epidermal keratinocytes and to a lesser degree by resident macrophages, Langerhans cells and fibroblasts in the dermis [5]. IL-6 is readily detected in cutaneous wounds [6], and in the supernatant of keratinocyte cultures subjected to in vitro wounding [7]. High levels of IL-6 have been associated to a number of skin pathologies, while mice genetically deficient in IL-6 (*Il6−/−*) display significantly delayed wound healing compared to wild-type animals [8].

A second major regulator of wound healing is the transforming growth factor (TGF)-β1 a growth factor that regulates cell proliferation, adhesion, migration and differentiation, and finally extracellular matrix deposition [9]. TGF-β1 has been shown to act as a mitogen and chemoattractant for fibroblasts and as inducer of myofibroblast differentiation [10]. Treatment with exogenous TGF-β1 has been shown to improve the healing process [11].

Corticotropin-Releasing Hormone or Factor (CRH or CRF), a 41-amino acid peptide and the major activator of the hypothalamic–pituitary–adrenal axis (HPA), has potent immunomodulatory properties [12] [13]. In vitro studies have shown that CRH modulates the secretion of the proinflammatory cytokines IL-1, IL-2, and IL-6 [14] [15] [16]. The development of the *Crh*-deficient (*Crh−/−*) mouse [17] has provided important information on the role of endogenous CRH in the activation of the immune system and the inflammatory response [18] [19]. *Crh−/−* mice exhibited significantly diminished inflammatory response in two experimental models of localized inflammation, carrageenin [20] and turpentine [21], while in preliminary studies we have found that *Crh−/−* mice have accelerated wound closure (unpublished data).

CRH and its receptors (CRF_1_, CRF_2_) are expressed in many peripheral tissues and organs including skin [22] where it has dual activity, direct proinflammatory and indirect anti-inflammatory [23] [24] [25]. In the human skin, CRF_1_ and particularly the CRF_1_alpha isoform, is the major receptor subtype expressed in both epidermal and dermal compartments, whereas CRF_2_ is detected predominantly in dermal structures. In rodent skin, both CRH receptors are expressed with CRF_1_ the predominant form in the keratinocytes and CRF_2_ in the panniculus carnosus [22]. Human normal or cancer skin cell lines express the *Crh* transcript, while in the mouse skin CRH has been suggested to derive from nerve endings [22]. CRH inhibits the proliferation of normal neonatal keratinocytes [22], whereas CRH-induced activation of the pro- and anti-inflammatory cytokines IL-6 and IL-11 and inhibition of IL-1β release from human keratinocytes was shown [26]. Furthermore, it has been hypothesized that skin CRH mediates the activation of the tissue response to local stressors, including inflammation and injury [27] [28]. Immunoreactive CRH has been identified in fibroblasts, monocytes, and endothelium of inflamed tissues [29] but its specific effects in dermal fibroblasts have not been studied.

Aim of the present work was to examine the presence of the CRH system (ligand and receptors) in murine skin fibroblast and evaluate its effect on several parameters of these cells. For this purpose we have studied the expression of CRH and its receptors in murine skin fibroblast, the effects of CRH on fibroblast proliferation, apoptosis and migration and its specific effect on the production of two major factors affecting wound healing that of IL-6 and TGF-β1. For this purpose, we have used fibroblasts isolated from the skin of wildtype (*Crh+/+)* and *Crh* deficient (*Crh−/−*) mice as well as from normal human foreskin.

## Results

### Expression of CRH and functional CRF_1_ and CRF_2_ receptors in murine fibroblasts

**Figure 1:**
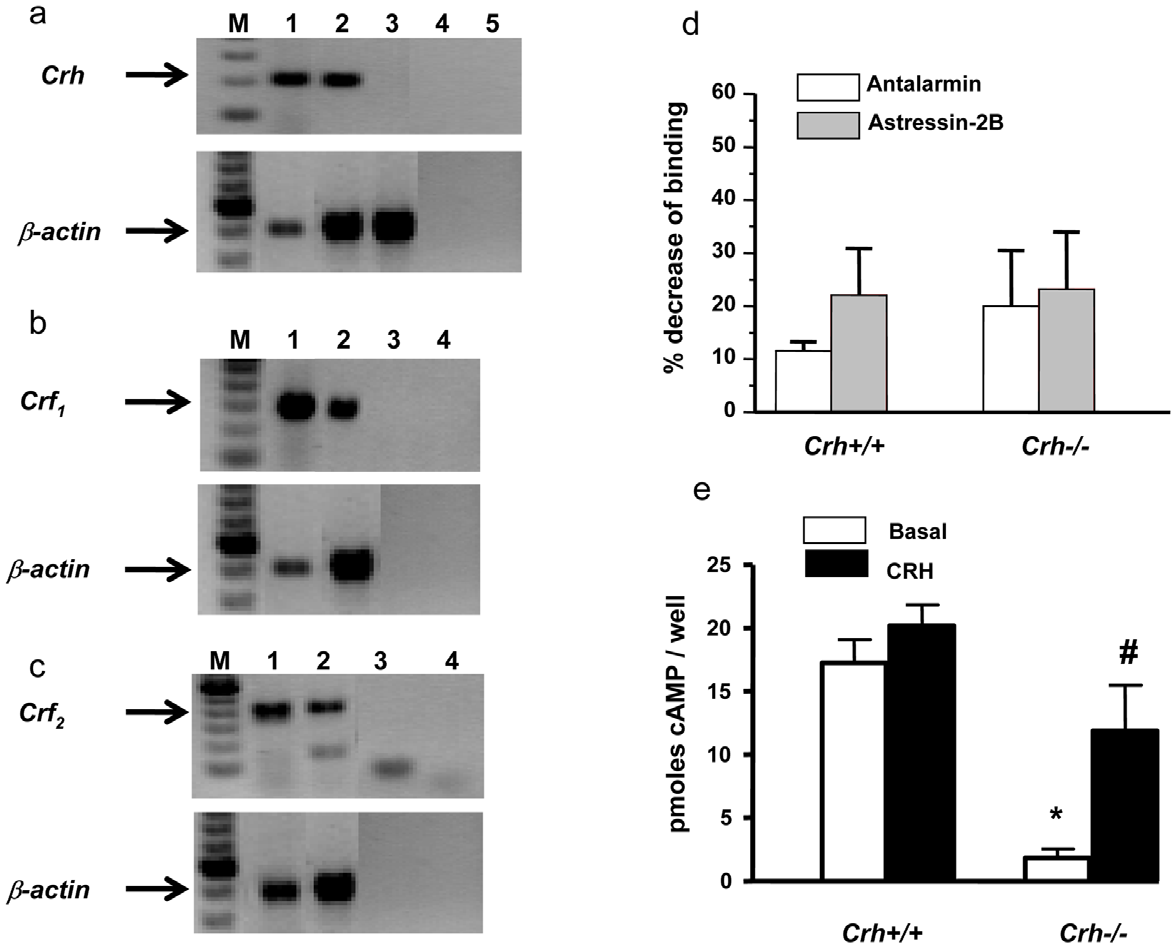
Mouse dermal fibroblasts express Crh and its receptors. (a) Total RNA isolated from whole brain from *Crh+/+* mice (lane 1), NMF isolated from *Crh+/+* mice (lane 2) and NMF isolated from *Crh−/−* mice (lane 3) was subjected to RT-PCR for evaluation of *Crh* expression. Lane 4 represents a sample without RT-enzyme and lane 5 represents a sample without template. (b) *Crf1* mRNA expression in NMF isolated from *Crh+/+* mice (lane 2). Lane 1 represents *Crf1* mRNA expression isolated from whole brain, while lane 3 represents a sample without RT-enzyme and lane 4 a sample without template. (c) *Crf2* mRNA expression in NMF isolated from *Crh+/+* mice (lane 2). Lane 1 represents *Crf1* mRNA expression isolated from heart, while lane 3 represents a sample without RT-enzyme and lane 4 a sample without template. (d) Effects of antalarmin and astressin-2B on specific [^125^I] Tyr0-sauvagine binding to CRF_1_ and CRF_2_. Membrane homogenates from *Crh+/+* and *Crh−/−* fibroblasts were assayed for specific binding with [^125^I] Tyr0-sauvagine, as described in Materials and Methods. The bars represent the % decrease of specific binding. The mean ± SEM values are from 3 independent experiments, each performed with duplicate determinations. (e) Effect of CRH on cAMP accumulation in *Crh+/+* and *Crh−/−* fibroblasts. Stimulation of cAMP accumulation by CRH was performed as described in Materials and Methods in intact cells. The mean ± SEM values are from 3 independent experiments, each performed with duplicate determinations. * represents statistical difference (P<0.05) between genotypes exposed to the same treatment and # represents statistical difference (P<0.05) between different treatments in the same genotype.

*Crh* mRNA expression was evaluated in murine fibroblasts isolated from newborn wildtype (*Crh+/+*) mice. A *Crh* transcript of identical molecular size to that of brain *Crh* was found in fibroblasts, as expected, while no similar band was detectable in *Crh*−/− fibroblasts (Fig. 1a). Furthermore, IR-CRH was measurable in the culture media of *Crh+/+* fibroblasts at concentrations of 0.8±0.21 ng/µg of total protein. We also assessed the expression of both *Crf_1_* and *Crf_2_* in fibroblasts isolated from *Crh+/+* mice. Both transcripts were detectable in more or less similar levels (Fig. 1b, 1c). To characterize the CRH receptors present in murine fibroblasts, we determined their binding capacity as well as their ability to stimulate cAMP accumulation. As shown in figure 1d treatment of membrane homogenates from *Crh+/+* fibroblasts with the CRF_1_- and CRF_2_-selective antagonists, antalarmin and astressin 2B, respectively, decreased ^125^I-Tyr^0^-sauvagine binding suggesting the presence of both (CRF_1_ and CRF_2_) proteins, in these cells. Interestingly, treatment with CRH at the saturating concentration of 250 nM was unable to significantly increase the already elevated basal cAMP levels in these cells (Fig. 1e) suggesting that endogenous CRH had saturated both receptors expressed in fibroblasts, and had, maximally, stimulated the CRF receptor-mediated cAMP accumulation. To confirm the role of endogenous CRH we assessed the expression, binding capacity, and CRH-stimulated cAMP accumulation of both types of CRH receptors in fibroblasts isolated from *Crh−/−* newborn mice. We found that both *Crf_1_* and *Crf_2_* transcripts and proteins (measured by RT-PCR and FACS analysis), were present in the *Crh−/−* mice at levels similar to those of *Crh+/+* fibroblasts. Treatment of *Crh−/−* fibroblasts with either antalarmin or astressin 2B reduced ^125^I-Tyr^0^-sauvagine binding confirming the presence of specific CRH binding sites (Fig. 1d). Interestingly, in contrast to *Crh+/+* fibroblasts, treatment of *Crh−/−* fibroblasts with CRH significantly increased cAMP accumulation at levels reaching the basal or CRH-stimulated ones in *Crh+/+* fibroblasts confirming the role of endogenous CRH (Fig. 1e).

### Effect of endogenous CRH on the proliferation and apoptosis of murine fibroblasts

**Figure 2:**
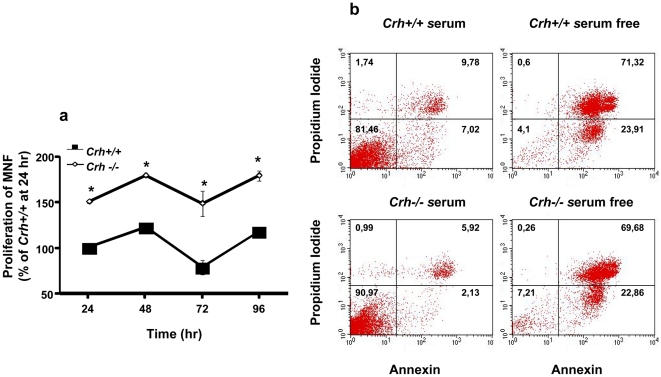
Proliferative response of Crh+/+ and Crh−/− fibroblasts. (a) Fibroblasts isolated from *Crh+/+* and *Crh−/−* dermis plated at an initial density of 8×10^3^ cells/well for four days. * represents statistical difference (P<0.05) between genotypes. (n = 4 wells/treatment/experiment, at least 5 independent experiments). (b) Fibroblasts isolated from both genotypes cultured at an initial density of 10^5^ cells/well in serum supplemented or serum free medium for 30 hr and apoptosis was measured by staining cells with Annexin V/Propidium Iodide and flow cytometry. The experiment was performed three times, with representative results of one experiment shown here.

To assess the biological significance of CRH in murine dermal fibroblasts, we compared the proliferation rates of *Crh−/−* and *Crh+/+* fibroblasts by performing MTT and thymidine incorporation methods. Both types of assays showed that basal proliferation rate of *Crh−/−* fibroblasts was much higher compared to *Crh+/+* fibroblasts suggesting that the endogenous CRH exerts a suppressive effect on fibroblast proliferation (Fig. 2a).

To assess the possibility that the suppressive effect of CRH on fibroblast proliferation also involve parallel effects on their apoptosis we also examined the effect of endogenous CRH on apoptosis. Thus, we examined apoptosis in *Crh+/+* and *Crh−/−* fibroblasts subjected to serum free starvation. Measurement of Annexin V thirty hrs following serum starvation showed no difference in the apoptosis levels between the two genotypes suggesting that endogenous CRH has no effect on apoptosis (Fig. 2b).

### Effect of endogenous CRH on murine fibroblast migration

**Figure 3:**
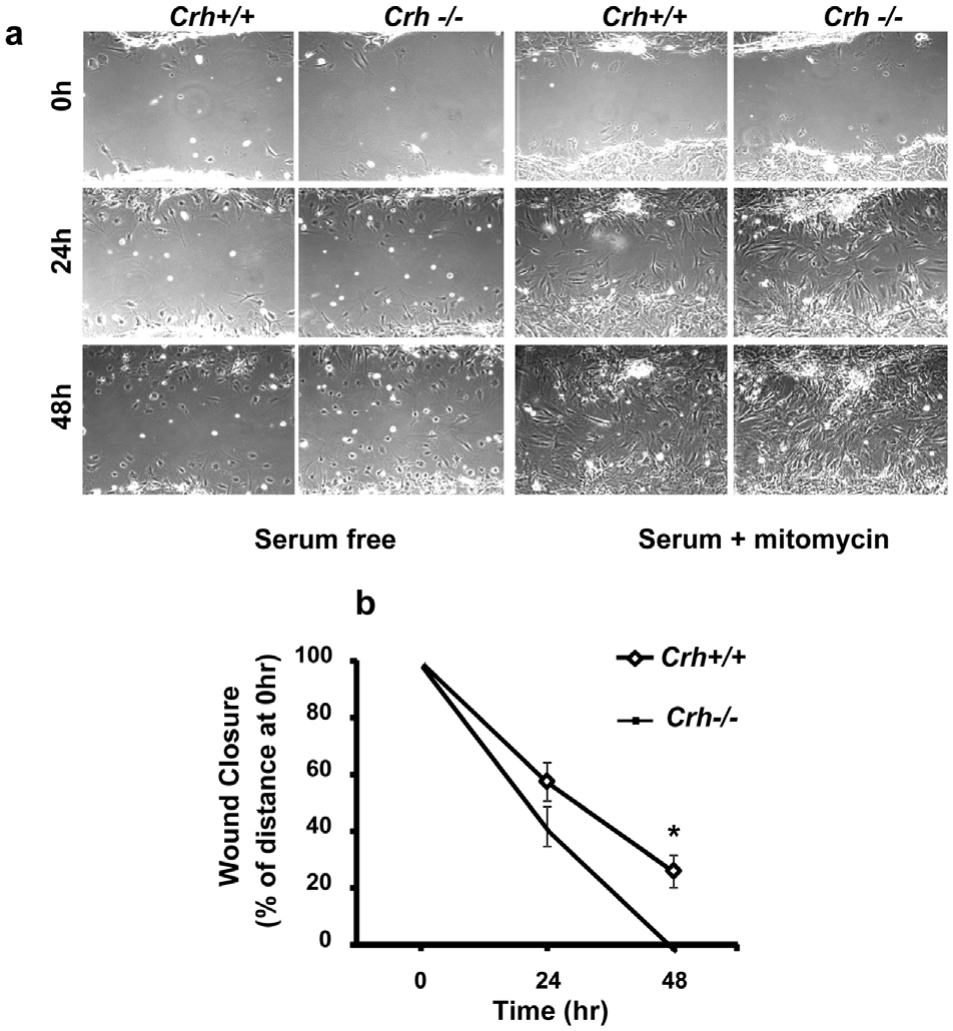
Enhanced migratory response of Crh−/− fibroblasts. Fibroblasts isolated from *Crh+/+* and *Crh−/−* dermis were allowed to attach on the dish. A clear space was produced in the confluent monolayer, cultured in serum free or serum treated with 10 µg/ml mytomycin and the wounded fibroblast layer was photographed immediately and 24 and 48 h after wounding. The distance of migration from the original borders was determined and is indicated (b) as a percentage of the original distance in a representative experiment. * represents statistical difference (P<0.05) between genotypes. (n = 3 wells/treatment/experiment, at least 4 independent experiments).

To determine a possible effect of CRH on the migratory capacity of fibroblasts *in vitro* we have used a scratch-wounding assay. Two types of experiments were performed in order to minimize cell proliferation for the migration assay to be valid: In the first set, the cells were pre-incubated for 2 hr with mitomycin in serum supplemented conditions before the scratch assay was performed while in the second set the cells were cultured in serum free conditions. As shown (Fig. 3a and b) within 24 hrs from the induction of the wound, the number of *Crh−/−* cells migrated into the wounded area was significantly higher compared to that of *Crh*+/+ cells suggesting that the endogenous CRH exerts a suppressive effect on the migration of fibroblasts.

### Effect of endogenous CRH on IL-6 and TGF-β1 production by murine fibroblasts

**Figure 4:**
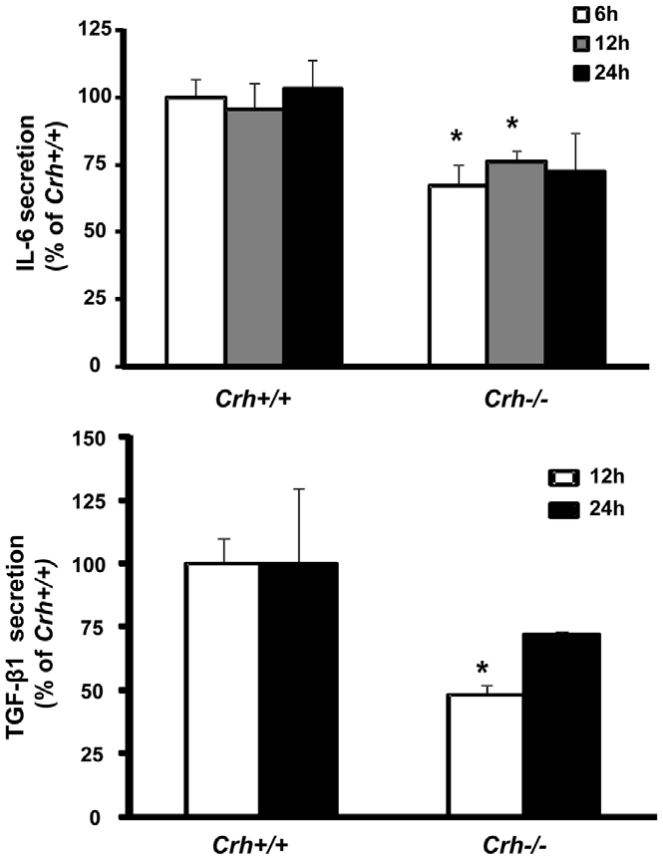
IL-6 and TGF-β1 production from Crh+/+ and Crh−/− fibroblasts. Primary dermal fibroblasts isolated from *Crh+/+* and *Crh−/−* mice were cultured in 24-well plates (10^5^ cells/well). Supernatants were collected and analyzed by ELISA for IL-6 (a) or TGF-β1 (b). * represents statistical difference (P<0.05) between genotypes (n = 3 wells/condition/experiment, at least 3 independent experiments).

Basal IL-6 and TGF-β1 secretion was significantly lower at all time points studied, in the media of *Crh−/−* fibroblasts (Fig. 4a and 4b, respectively) suggesting that endogenous CRH exerts a pro-inflammatory effect on fibroblast a phenomenon described already for several types of immune cells.

### Effects of endogenous CRH on human fibroblasts

**Figure 5:**
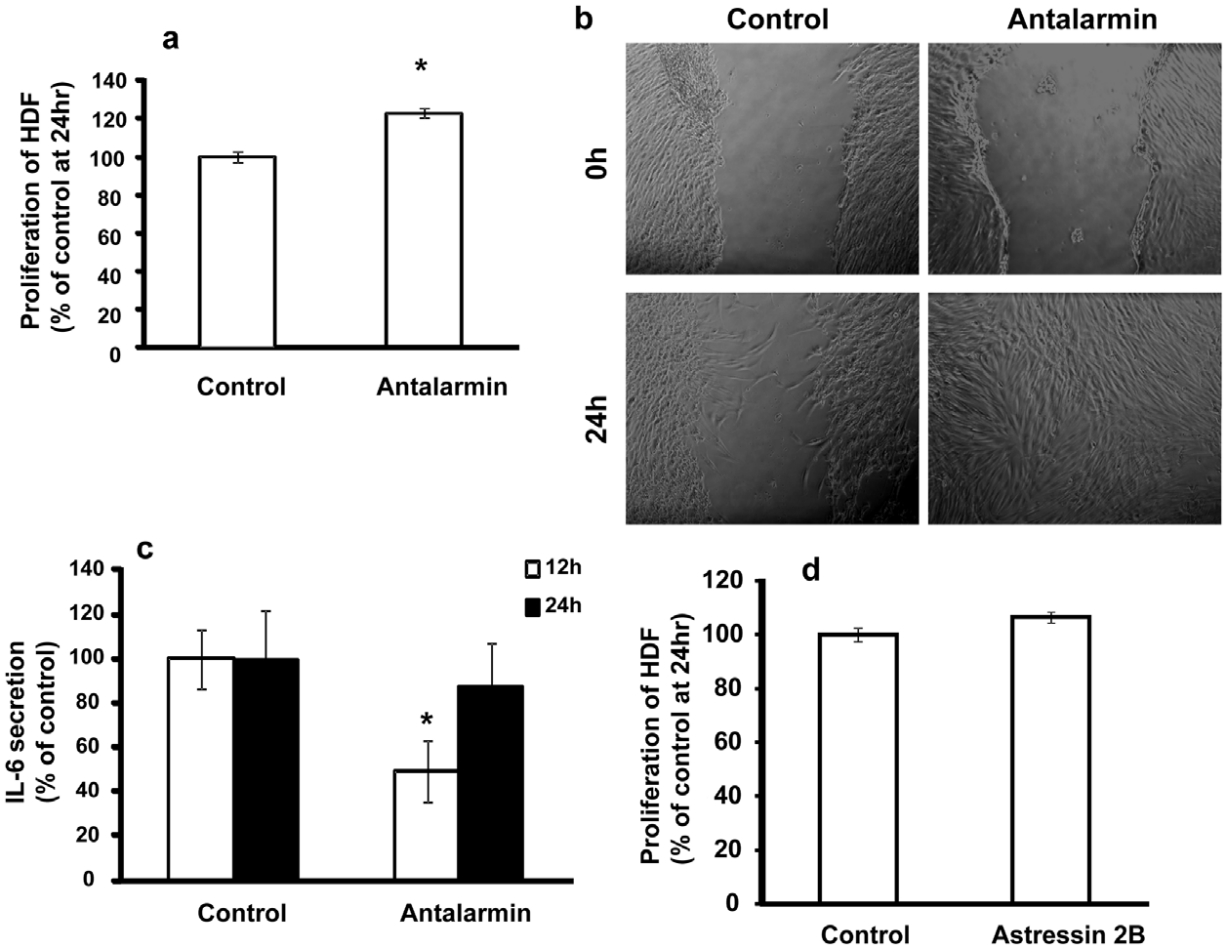
Effect of the CRH antagonists in human dermal fibroblasts. (a) HDFs isolated from human foreskin were cultured in 96-well plates (8,000 cells/well) in the presence of 100 nM antalarmin or ethanol (control). Proliferation was measured using either MTT or thymidine incorporation. *: represents statistical difference (P<0.05) between treatments (n = 4 wells/treatment/experiment, at least 3 independent experiments). (b) HDFs were allowed to attach on the 100 mm dish. A clear space was produced in the confluent monolayer, cultured in serum free or serum treated with 10 µg/ml mytomycin and the wounded fibroblast layer was photographed immediately and 24 h after wounding (n = 3 wells/treatment/experiment, at least 3 independent experiments). (c) HDFs were cultured in 24-well plates (10^5^ cells/well) in the presence of 100 nM antalarmin or ethanol (control). Supernatants were collected and analyzed by ELISA. * represents statistical difference (P<0.05) between treatments (n = 3 wells/condition/experiment, at least 3 independent experiments). (d) HDFs were cultured in 96-well plates (8,000 cells/well) in the presence of 1000 nM astressin 2B or ethanol (control). Proliferation was measured with MTT, (n = 4 wells/treatment/experiment, at least 3 independent experiments).

Human primary fibroblasts have been shown to express CRH as well as the CRF_1_ receptor [22]. To assess the relevance of our findings in human cells, we isolated fibroblasts from juvenile human foreskin and cultured them in the presence of the CRF_1_ specific antagonist anatalarmin or vehicle. Exposure of human foreskin fibroblasts to antalarmin (100 nM) resulted in an increase of their proliferation (Fig. 5a) and migration rate (Fig. 5b) while it suppressed IL-6 production (Fig. 5c). In contrast, treatment of the cells with Astressin 2B (a specific CRF_2_ antagonist) had no effect on either their proliferation or migration rates. The findings in human fibroblasts are similar with the above described mouse data suggesting that a significant degree of similarity exists in the pathways mediated by endogenous CRH in human and mouse primary fibroblasts function.

### Effects of neutralization of IL-6 and TGF-β1 on the proliferation and migration rate of dermal fibroblasts

To determine the impact of the lower IL-6 and TGF-β1 secretion on the higher proliferation and migration rate of *Crh/−/−* fibroblasts we immunoneutralized either of the factors using specific antibodies in cultures of *Crh+/+* mouse and human fibroblasts. We found that blockade of either IL-6 or TGF-β1 did not alter either the migration or the proliferation rate of both mouse and human fibroblasts (data not shown).

## Discussion

The present study examines the role of endogenous CRH (i.e. CRH produced within the skin) on the biology of dermal fibroblasts. For this purpose we have used fibroblasts isolated from the skin of wildtype (*Crh+/+*) and *Crh* deficient (*Crh−/−*) mice as well as from normal human foreskin. To our knowledge, this is the first report on the expression of the CRH gene in murine fibroblasts and on its role in several key functions of these cells. Our data suggest that locally produced CRH plays an important role in the biology of murine and human skin fibroblast, an effect inferred from our observations in *Crh+/+* and *Crh−/−* mice as well as from normal human foreskin.

More specifically, we first demonstrate that murine fibroblasts isolated from neonatal skin express CRF, in contrast to the reported lack of expression in fibroblasts isolated from the skin of adult or adolescent mice [22] [30]. This discrepancy in the expression could be attributed to the different source of fibroblasts, or age, or developmental stage. We also found that both types of CRH receptors, CRF_1_ and CRF_2_ are expressed in dermal fibroblasts and that they are fully functional since they are able to bind the ligand in a specific manner and that their activation results in cAMP production (Fig. 1). These data are in agreement with previous reports showing that human dermal fibroblasts also express CRF_1_ receptor and its activity is regulated by exogenously added CRF via activation of cAMP and IP3 [31] [32]. It should be noted here that cAMP accumulation experiments were carried out in intact cells which most likely retain their ability to produce CRH. Thus, it seems that the endogenous CRH produced and secreted from wildtype fibroblasts saturated the CRF_1_ and CRF_2_, expressed in these cells, and maximally stimulated the CRF receptor-mediated cAMP accumulation. To further confirm this hypothesis we treated wildtype fibroblasts with specific antagonists for CRF_1_ and CRF_2_ (antalarmin and astressin 2B, respectively), that resulted in decreased cAMP accumulation in either case (data not shown). This hypothetical model might explain the lack of an additional effect of exogenously added CRH on cAMP accumulation in *Crh+/+* fibroblasts.

Subsequently, we describe accelerated basal proliferation as well as migration rate in primary *Crh−/−* compared to *Crh+/+* fibroblasts with a simultaneous compromised IL-6 and TGF-β1 secretion. These findings suggest specific effects of endogenous CRH at several independent indices of fibroblast biology both under basal conditions as well as after the induction of in vitro wounding. Previously published elegant studies have shown that, although the CRH/CRF receptors system is expressed in both human and mouse skin and it appears that it plays an important role in cutaneous biology of both, there are species-specific differences including the predominant receptor type expressed [22]. To our surprise, pharmacological blockade of CRH in primary human dermal fibroblasts induced a phenotype very similar to what we have found in the *Crh−/−* fibroblast.

In previous studies, we found paradoxically altered IL-6 levels in *Crh−/−* mice following turpentine-induced abscess formation, although to the opposite direction of the one found in the fibroblasts [21]. Tissue-specific effects related to the receptor subtype expression, the predominant signaling pathway operating as well as additional factors expressed in dermal fibroblasts may provide an explanation for the above findings. The interaction between IL-6 and TGF-β in dermal fibroblasts has not been completely clarified. In other tissues, such as the lung, downregulation of IL-6 resulted in abrogation of TGF-β1–induced fibroblast proliferation [33], while TGF-β1 itself increased endogenous IL-6 levels [34]. Similarly, TGF-β1 expression was induced in *Il6−/−* fibroblasts treated with recombinant IL-6 [35].

In conclusion, our experimental data obtained from *Crh−/−* and wildtype mice as well as from human foreskin fibroblast suggest that the endogenously produced CRH plays an important physiological role in several parameters of skin fibroblast function. In essence, the primary effect of endogenous CRH appears to be suppressive and pro-inflammatory. Our current data add on previous reports on the physiological significance of stress neuropeptides on the function of skin fibroblasts and wound healing. Furthermore, our results may have significant implications in the design of new therapeutic antagonists targeting dermal fibroblasts in wound healing.

## Materials and Methods

### Ethics statement

All animal experiments were approved by the Animal Committee of the Medical School of the University of Crete and the Veterinary Department of Heraklion Prefecture. The Animal Facility of the Medical School of the University of Crete has been licensed by the Veterinary Department of Heraklion Prefecture. All experiments with human fibroblasts cultures were approved by the Ethics Committee of the University Hospital of Heraklion (Official name of the Committee). A written consent was obtained from all participants involved in the study.

### Isolation and culture of murine and human dermal fibroblasts

Primary murine dermal fibroblasts were isolated from the skin of *Crh+/+* and *Crh−/−* neonatal mice, of 129xC57BL/6 genetic background, (derived from *Crh+/+*×*Crh+/+* and *Crh−/−*×*Crh−/−* matings, respectively), as previously described with some modifications [2]. Briefly, newborn *Crh+/+* and *Crh−/−* mice (0–48 hours old) were sacrificed by decapitation, treated for 10 min in a povidine–iodine bath, followed by a 10 min 70% ethanol bath and the whole skin was removed. Excised mouse skin was placed in Petri dishes with the dermis side down, in Dulbecco-PBS buffer (Invitrogen, Grand Island, N.Y., USA) containing 10 mg/ml dispase II (Sigma, Germany) and incubated at 4°C, overnight. Isolated dermal tissue was then cut and placed in tissue culture plates. Fibroblasts were cultured in DMEM (Gibco-BRL, Gaithersburg, MD) with 10% fetal bovine serum (Gibco-BRL) and 1% penicillin/streptomycin (Gibco-BRL) after the tissue had been attached to the plate. The fibroblasts were checked for their spindle shaped morphology and identified by immunostaining with an anti-vimentin goat anti-mouse antibody (a kind gift by Professor Christos Stournaras [36]) or anti-pancytokeratin monoclonal mixture antibody (Sigma) for keratinocyte contamination (data not shown). The cells were then re-plated twice to deplete the culture from residual keratinocytes that do not survive re-plating in 10% FBS-DMEM [37]. In all experiments, *Crh+/+* and *Crh−/−* cells at identical passage numbers (<5) were used. Human fibroblast cultures were established, using the same protocol, from explants of foreskin obtained at the time of circumcision for unrelated problems.

### Fibroblast proliferation rates

The proliferation rate of murine and human fibroblasts was determined with the MTT and the thymidine incorporation methods [38]. For both methods, fibroblasts from either species were cultured at an initial concentration of 8,000 cells/well, in flat-bottomed 96-well plates (Corning, New York, NY, USA) in serum-supplemented medium. The cells were left to rest overnight and then exposed to the tested substances or their vehicles in serum and serum free conditions, as described in the Results. For the MTT method, at the end of the incubation period, MTT (Sigma) was added to each well at a final concentration of 0.5 mg/ml and cells were incubated for an additional 4 h at 37°C. Crystals were dissolved using DMSO (Sigma), and the optical density was read on a Microplate Reader, Biorad. For the thymidine incorporation method, at the end of the incubation period, [^3^H] Thymidine (Amersham Biosciences, Munich, Germany) was added to each well at a final concentration 1 µCi/well, and cells were incubated for an additional 24 h. Cells from each well were harvested onto glass-fiber filters (Whatman, England) using a semi-automatic harvester (Skatron Instruments AS, Lier, Norway). The incorporated radioactivity was counted using a β-counter (Perkin Elmer, Foster City, CA).

### Fibroblast apoptosis rates

Murine *Crh*+/+ and *Crh*−/− fibroblasts were cultured in 6-well plates. Twenty four hours later the medium was aspirated and replaced either with serum supplemented or serum free medium. Apoptosis was quantified at several time points with annexin V-FITC and Propidium Iodide (BD Pharmingen, Franklin Lakes, NJ, USA) by following manufacturer's instructions. Briefly, detached by trypsin, cells were washed twice with cold PBS and then harvested in Binding Buffer (provided in the kit). Annexin V-FITC (4 µl) and PI (3 µl) was added in 100 µl/10^5^ cells and incubated for 15 min at RT in the dark. At the end of the incubation period, 400 µl of BB was added and the cells were analyzed at a Beckton-Dickinson FACSArray apparatus (BD Pharmigen) using the CELLQuest software.

### Isolation of total RNA and Semi quantitative RT-PCR

Total RNA preparation and RT-PCR was performed as we have previously described [39]. The primers for the mouse specific mRNAs were obtained from VBC Biotech (Vienna, Austria). The sequence of the primers used and the expected size of the corresponding products are listed below:

Primers Size of product (bp)

*Mouse*

β-actin sense: 5′-TCAGAAGGACTCCTATGTGG-3′ 500

β-actin antisense: 5′-TCTCTTTGATGTCACGCACG-3′

CRF_1_ sense: 5′-GCCGCCTACAATTACTTCCA-3′ 310

CRF_1_ antisense: 5′-CGGAGTTTGGTCATGAGGAT-3′

CRF_2_ sense: 5′-CTGGTGGCTGCTTTCCTGCTTTTC-3′ 407

CRF_2_ antisense: 5′-ATGGGGGCCCTGGTAGATGTAGTCC-3′

CRH sense: 5′-AGCCCTTGAATTTCTTGCA-3′ 202

CRH antisense: 5′-AACACGCGGAAAAAGTTA-3′

Amplification of the cDNA was done using the conditions: 96°C for 3 min for denaturation, following by 40 cycles (25 cycles for β-actin) for 1 min at 94°C, annealing at 55–65 depending on the primers °C for 1 min, and extension at 72°C for 2 min. Reactions were completed after an additional 5-min extension at 72°C. The amplification reactions for all PCRs performed were analyzed on 1.3% agarose gels.

### IL-6 and TGF-β1

Murine and human fibroblasts were cultured at an initial concentration of 75,000 cells/well, in 24-well plates. The day of the experiment the media were replaced with fresh serum-free media containing vehicle or the tested substances, as indicated in the figure legends. Culture media were collected at the indicated, in the Results, time points and stored at −80°C until used. Human IL-6 levels were measured by chemiluminescence on Immulite 1000 [40]. Murine IL-6 and TGF-β1 levels were evaluated using commercial ELISA kits according to the instructions of the manufacturer (R&D Systems, Minneapolis, MN, USA). For the IL-6 and TGF-β1 immunoneutralization studies, the antibodies were purchased from R&D Systems and were used at concentrations of 1 µg/ml. Cells from both species were collected for protein extraction and measurement.

### CRH Peptide Enzyme Immunoassay (EIA)

Peptide in culture media was concentrated by a C-18 reverse phase column (Sep-Pak, Waters Associates, Milford, MA) after acidification in 2 vol 0.1 N HCI and centrifuged at 1500 rpm for 10 min. The supernatants were extracted by activated Sep-Pak cartridges, washed with 20 ml 0.1 N HCI eluted with 3 ml acetonitrile 80%-0.01% HCI, then dried under vacuum (Speed-Vac). The IR-CRH content of the Sep-Pak extracts was assayed by an EIA kit according to the manufacturer's instructions (Peninsula Laboratories, USA). The average IC50 of the assay was 0.5 ng/ml and the crossreactivity was 100% with mouse, human, and rat CRF, and 0% for Urocortins. Results were expressed as nanograms of IR-CRH per microgram of total cellular protein determined on whole cellular homogenates by the Bradford method.

### Fibroblast migration rate

Murine and human fibroblasts were cultured in 12-well plates until they reached confluency. The day of the experiment the media were replaced with fresh media containing vehicle or the tested substances and the scratch (in vitro wound) was performed as previously described [41] with a tip. Images were captured at several time points (indicated in the figure legends) and cell migration was quantified by measuring the distance with the program Image J (http://rsbweb.nih.gov/ij/) between two certain points on either side of the gap. For proper statistical evaluation, at least three measurements at different points were performed at each image.

### Binding assays

#### Harvesting cells and membrane preparation

Fibroblasts from *Crh+/+* and *Crh−/−* neonates were washed with PBS, at room temperature, briefly treated with PBS containing 2 mM EDTA (PBS/EDTA), and then dissociated in PBS/EDTA. Membrane suspensions were prepared as described previously [42]. ^125^I-Tyr^0^-sauvagine (PerkinElmer, Waltham, MA, USA) binding studies were performed as previously described [42]. Aliquots of diluted membrane suspension (50 µl) were incubated with 350–400 pM ^125^I-Tyr^0^-sauvagine with or without 500 nM Astressin-2B (a kind gift from Dr. Spiess), 1000 nM antalarmin (a gift from Dr. G. Chrousos) in a final volume of 0.1 ml. The mixtures were incubated at 20–21°C for 120 min and then filtered through glass fiber filters (934AH; Whatman) presoaked for 1 h in 0.3% polyethylenimine at 4°C. The filters were washed 3× with 0.5 ml of ice-cold PBS, pH 7.1, containing 0.01% Triton X-100 and assessed for radioactivity in a gamma counter (LKB Wallac 1275 minigamma, 80% efficiency).

### cAMP accumulation assays

Fibroblasts from both genotypes were plated in 6-well cell culture plates. When the cells became 95 to 100% confluent the medium was removed, and 500 µl of assay buffer was added [42]. After 1 hr incubation at 37°C, more buffer without (basal levels) or with 250 nM CRH, 1000 nM antalarmin, or 1000 nM astressin 2B was added to a total volume of 1000 µl, and the incubation was continued for 30 min at 37°C. At the end of the incubation, the buffer was removed. The cells were plated on ice and lysed with 1% trichloroacetic acid. Lysates were incubated on ice for 45 min, sonicated for 4 sec and stored at −80°C. Frozen lysates were thawed, centrifuged at 1700 g for 10 min at 4°C, and the supernatants were neutralized with 2N NaOH. Quantification of cAMP in the neutralized supernatants was performed using a competitive binding assay as described previously [42]. The amount of cAMP in each sample was determined by comparison with a standard curve of known concentrations of unlabeled cAMP.

### Statistical analysis

All values are expressed as the Mean ± SEM of data obtained from at least three independent experiments. Data were analyzed by Student's t-test and one-way analysis of variance (ANOVA) followed by *post-hoc* multiple comparison tests. Significance was accepted at p<0.05.
